# Evaluation of the effect of thermocycling on the trueness and precision of digitally fabricated complete denture bases

**DOI:** 10.1186/s12903-024-04636-5

**Published:** 2024-08-23

**Authors:** Ahmed Abd El-latif Zeidan, Mohamed Ahmed Helal

**Affiliations:** 1 Department of Removable Prosthodontics, Faculty of Dental Medicine, Badr University, Cairo, Egypt; 2 Department of Prosthodontics, Faculty of Dental Medicine, Al-Azhar University, Cairo, Egypt; 3 Faculty of Dentistry, Department of Prosthodontics, Al-Azhar University, Almokhyam Aldaem St., Nasr Road, Nasr City, Cairo 11884 Egypt

**Keywords:** Additive technique, CAD-CAM, Precision, Subtractive technique, Superimposition, Thermocycling, Trueness

## Abstract

**Background:**

While many denture base materials are currently available on the market, little data exists regarding their dimensional stability after exposure to the oral environment. This study aimed to evaluate the effect of thermocycling on the trueness and precision of milled, 3-dimensional (3D)-printed, and conventional digitally fabricated complete denture bases (CDBs).

**Methods:**

A completely edentulous maxillary stone model was scanned to generate a standard tessellation language (STL) file; this was imported into metal-milling-machine software (Redon Hybrid CAD-CAM metal milling machine, Redon, Turkey) to produce a metal model for fabricating 30 CDBs. These were divided into three groups (*n* = 10 in each) according to the construction technique: group 1, CAD-CAM milled CDBs; group 2, 3D-printed CDBs; and group 3, conventional compression molded CDBs. All CDBs were scanned after fabrication and evaluated before and after thermocycling using superimposition. The data were analyzed using a one-way ANOVA, Tukey’s post hoc test, and a paired t-test.

**Results:**

The level of trueness between the CAD-CAM milled, 3D-printed, and compression molded CDBs showed significant differences before and after thermocycling (*P* < 0.05). Group 1 showed the highest degree of trueness before and after thermocycling, group 3 exhibited a higher degree of trueness than group 2 before thermocycling, and group 2 had a higher degree of trueness than group 3 after thermocycling. There was a significant difference in the precision for each CDB type before and after thermocycling (*P* < 0.05).

**Conclusion:**

The trueness of the CAD-CAM milling system in complete denture (CD) fabrication is superior to that of the 3D printing and conventional compression molding systems before and after thermocycling. Thermocycling had a significant effect on the precision of all CDB types. The compression molding system in CD construction is the most negatively affected via thermocycling with regard to the measures of trueness and precision.

**Clinical trial number:**

Not applicable, no human participants were involved.

## Background

Most completely edentulous patients prefer complete dentures (CDs) due to their low invasiveness and cost-effectiveness. One of the critical factors affecting the quality of a complete denture base (CDB) is its degree of fitness to underlying tissue structures; CDBs that fit well provide better primary comfort and lower the risk of traumatic ulcers. The most significant aspect for good retention in CDBs is a tissue-congruent denture fit. The retention of CDBs has a marked effect on masticatory efficiency and speaking ability and, thus, on patients’ quality of life. Therefore, one of the key goals in CDB fabrication should be to achieve maximum tissue congruence [1, 2].

During the polymerization, cooling, and finishing operations, the polymethylmethacrylate (PMMA) denture base deforms linearly by 0.45 to 0.9%. Numerous investigations of production processes, denture base materials, and clinical protocols have been made to manage the extent of polymerization shrinkage. By injecting unpolymerized acrylic resin into the mold, researchers have tested the pressure injection molding technique to improve the dimensional accuracy of the compression molding process [3, 4].

Prior to the development of computer-aided design–computer-aided manufacturing (CAD-CAM) technology in the field of dental prosthetics, resin shrinkage during polymerization inhibited the correspondence between CDBs and the tissues that support dentures. Deformities generated on CDBs by this shrinkage thus had a negative effect on CD retention and fit [5].

CAD-CAM technologies have been introduced and undergone several developments over the past few years [6, 7]; these fall into two categories: milling and three-dimensional (3D) printing. Milling technology depends on a subtractive process, while 3D printing depends on an additive process [8, 9].

Milling technology is advantageous for both professionals and patients. Five appointments are usually required for CD construction, which is time-consuming for both technician and dentist. On the other hand, milled dentures can be delivered in as few as two sessions, significantly reducing the time requirements. In addition, patient records are digitally stored; thus, if a patient’s dentures are damaged or destroyed, an accurate replacement prosthesis can be rapidly created without the need for additional clinical documentation [10, 11]. However, this technology presents some drawbacks, including inevitable material loss during milling; high equipment maintenance costs; significant time wastage during the production process; and the dependence of restoration accuracy on the size of the milling burs and the wear, tear, and short lifetime of the milling tools [12–14].

3D printing technology allows for the production of necessary models and prostheses using a small amount of material. It relies on additive manufacturing and constructs objects using a layer-by-layer process; furthermore, the capacity for continuous production considerably promotes clinical efficiency. Due to these advantages, the use of 3D printers in dentistry has substantially increased since their creation [15–17].

In dentistry, 3D printing is currently utilized to create diagnostic models, orthodontic treatment plans, and implant surgical guides and to fabricate specialized orthodontic equipment. However, the value and precision of existing procedures are insufficient to create models for prostheses, which is considered a limitation of 3D technology; in addition, the accuracy with which an object is constructed depends on the efficiency of the 3D printer and the 3D-printed liquid [18–21].

In one study, scholars evaluated the dimensional accuracy of CAD-CAM milled, 3D-printed, and conventional compression molded CDBs [6], though they did not compare the effect of thermocycling on the trueness of the CDBs. Moreover, there is limited data available in the literature regarding the effect of thermocycling on the precision of different CDB types. Therefore, this study aimed to evaluate the effect of thermocycling on the trueness and precision of CAD-CAM milled, 3D-printed, and conventional compression molded CDBs.

The null hypotheses of this study were as follows: the differences in (i) the trueness between CAD-CAM milled, 3D-printed, and conventional compression molded CDBs and (ii) the precision within each CDB type before and after thermocycling are insignificant.

## Methods

A power analysis was designed to apply statistical tests to the null hypothesis stating that there is no difference in the dimensional accuracy between the groups. Based on an alpha level of 0.05, a beta level of 0.05 (i.e., power = 95%), an effect size of 0.759, and calculations from the study by Kalberer et al. [7], the predicted sample size was 30 (10 samples/group). The sample size calculation was performed using G*Power, version 3.1.9.7 [8].

In total, 30 maxillary CDBs were investigated herein. These were divided into three groups based on the processing technique and resin composition (*n* = 10 in each): group 1 (G1), CAD-CAM milled CDBs; group 2 (G2), 3D-printed CDBs; and group 3 (G3), conventional compression molded heat-cured PMMA CDBs.

A ready-made completely edentulous maxillary stone model was employed for this study [6, 10]. The reference CAD standard tessellation language (STL) file was created by scanning the master stone model with an optical extraoral 3D scanner (Dentsply Sirona inEos X5, Germany).

A preview of the 3D cast model was sent for approval after it had been virtually created using CAD software (ExoCad, Chairside Cad 2.3 Matera, Germany), and the manufacturing of the edentulous metal master cast was approved. Using the scan data from the reference CAD model file, a CAD-CAM metal milling machine (Redon Hybrid, Turkey) and a CAD metal disc (Vsmile titanium grade 5 98*25 mm universal, China) were used to construct the titanium metal cast. Burs with diameters of 2, 1, and 0.5 mm were used in the milling process of the metal model. One set of milling burs was used for milling a single block to maintain the same conditions during the milling process [16].

The metal master cast was then finished and polished using standard techniques. An optical extraoral 3D scanner was used to scan the reference CAD master metal model, and the resulting data was recorded as an STL file. During the scanning procedures, a scanning spray (Telescan spray-white, Germany) was utilized to eliminate the metal model’s reflections [14].

### Processing of CAD-CAM milled CDBs (G1)

Ten CAD-CAM milled CDBs were fabricated as follows [14, 18].

The STL file of the scanned metal model was exported to CAD software. After obtaining the scan data, the STL file was imported into ExoCad design software, where the anatomical landmarks were automatically detected and indicated [14].

After its approval, the virtual CDB design was exported to a CAM laboratory milling machine (Dentsply Sirona inLab MC X5, Germany). Prepolymerized CAD-CAM standard single-linked PMMA acrylic pucks (AvaDent Digital Dental Solutions HQ, USA) were then milled into CDBs [18].

The outline of the CDB was produced using a bur with a maximum diameter of 2.5 mm, while the finer details were implemented using a bur with a minimum diameter of 1 mm. The manufacturer’s recommendations were followed, and the milling operation was set to 5-axis machining to accurately produce the fine details and carried out in moist conditions to prevent overheating [15].

### Processing of 3D-printed CDBs (G2)

Ten 3D-printed CDBs were fabricated based on the previously described virtual design as follows [9].

The designed CDBs were exported as STL files to a 3D printer (Wanhao desktop, China) and printed according to Digital Light Processing (DLP) technology using photopolymerized 3D-printed liquid (Harz Labs, Moscow, Russian Federation) [9].

Before printing, the Harz Labs liquid (methacrylated oligomers, methacrylated monomers, and photoinitiator) was placed in the supply chamber of the 3D printer and shaken for roughly 5 min. The CDBs were printed with a layer thickness of 100 μm/layer at an orientation of 45°. The layers were cured successively and bonded together to fabricate the desired CDB; the bonding of the layers resulted from the material’s inherent self-adherence. To remove any extra material, the finished printed CDBs were rinsed twice in isopropyl alcohol (99% concentration), first for 3 min and then for 2 min. Thereafter, they were placed in an ultraviolet (UV) light-curing box (Anycubic wash and cure light box, Shenzhen, China) for an additional 15 min of polymerization [9].

### Processing of conventional compression molded heat-cured CDBs (G3)

Ten conventional CDBs were processed using the conventional compression molding technique on heat-cured PMMA resin (Vertex-Dental B.V. headquarters, the Netherlands), described as follows [10].

For this group of CDBs, a specially designed stone mold was constructed using the reference CAD metal model. According to the manufacturer’s recommendations, the heat-cured PMMA powder and liquid were mixed in a glass jar in a polymer-to-monomer ratio of 3:1 to reach the dough stage. The two halves of the dental flask were closed and placed under hydraulic compression, and pressure was slowly applied to the flask to allow the resin dough to flow throughout the mold space [10].

The flask was then immersed in a thermostatic temperature-controlled water bath for curing. The PMMA was cured and polymerized over a long curing cycle according to the manufacturer’s instructions. In the curing cycle, the flask was placed in water at 70 °C for 9 h, after which it was removed from the curing bath and cooled slowly for 30 min to room temperature. The mold was then opened, the CDBs were removed, and excess acrylic was trimmed [10].

After constructing all the CDB specimens, they were finished using tungsten carbide acrylic burs (Edenta AG, Au, Switzerland) and silicon carbide papers. The specimens were further polished using rubber acrylic burs (Edenta), pumice (Shera, Lemförde, Germany), and rouge (Dialux, Lüdenscheid, Germany). Only one surface was polished, while the interior surface was left untouched to simulate oral surfaces to the greatest extent possible [11].

### Trueness evaluation

The intaglio surfaces of the 30 CDBs were scanned using an optical extraoral 3D scanner (Dentsply Sirona inEos X5, Germany) and stored as STL files to assess the trueness of the various groups of CDBs. The superimposition evaluation technique was performed, described as follows [21].

Surface matching software (Geomagic Control X, 3D Systems, Canada) was used to superimpose the STL files of the scanned intaglio surfaces of the 30 dentures on the STL file of the scanned reference model, using a best-fit alignment to assess the trueness and congruence of the CDs relative to the model. The vertical distances between the superimpositions were calculated using the software. The findings were depicted as color maps, with yellow to red denoting CDB impingement on the cast, blue indicating separation between the CDB and cast, and green denoting contact between the CDB and cast. The closer the value is to 0, the more accurate and adaptive the fabrication technique is.

### Precision evaluation

The master cast surface was divided into five functionally relevant sections (anterior ridge crest, posterior ridge crest, maxillary tuberosity, palatal vault, and posterior palatal seal) to assess the region-specific mismatches and evaluate the precision of the different groups of CDBs.

### Thermocycling

A thermocycling protocol simulating 6 months of intraoral use was applied to all specimens. The dentures were immersed alternately in deionized water at 5 and 55 °C for 5000 cycles [5]. After thermocycling, the scanning and matching procedures were repeated, following the mentioned protocol.

### Statistics

The normality and distribution of the data were assessed through normality tests (Shapiro-Wilk and Kolmogorov-Smirnov), with all data showing a parametric (normal) distribution. The data were analyzed using an ANOVA test to determine whether significant differences existed between the means of the trueness of the tested groups. In addition, Tukey’s post hoc (TPH) test was used for pairwise comparisons between the groups at the selected level of probability (*P* < 0.05). However, a paired t-test was employed to study the effect of thermocycling on the precision at different sites within the same group at the chosen level of probability (*P* < 0.05), using Statistical Product and Service Solutions, version 20 (SPSS; IBM Corporation, NY, USA) for Windows and Graph Pad Prism, version 8 (Graph Pad Prism Company, USA).

## Results

### Overall denture fit

The informative statistical analyses of the different groups (pre- and post-thermocycling) using a one-way ANOVA are presented in Table 1.

**Table 1:** Means and standard deviations (mm) of the trueness of the tested groups, one-way ANOVA test, and the pairwise comparisons between tested groups (before and after thermocycling)

	Superimposition measurements	ANOVA test P-value	Tukey post hoc test	
	Mean ± SD (mm)			
			Pair-wise Comparisons	P-value
Group1 (Before thermocycling)	0.60 ± 0.023	0.0001*	Group1 vs. Group2	< 0.0001*
Group 2 (Before thermocycling)	0.96 ± 0.032		Group1 vs. Group3	< 0.0001*
Group 3 (Before thermocycling)	0.93 ± 0.024		Group2 vs. Group3	0.046*
Group 1 (After thermocycling)	0.67 ± 0.089	0.0001*	Group1 vs. Group2	< 0.0001*
Group 2 (After thermocycling)	1.06 ± 0.06		Group1 vs. Group3	< 0.0001*
Group 3 (After thermocycling)	1.56 ± 0.15		Group2 vs. Group3	< 0.0001*

*; Significant level at *p* ≤ 0.05

**Fig. 1:**
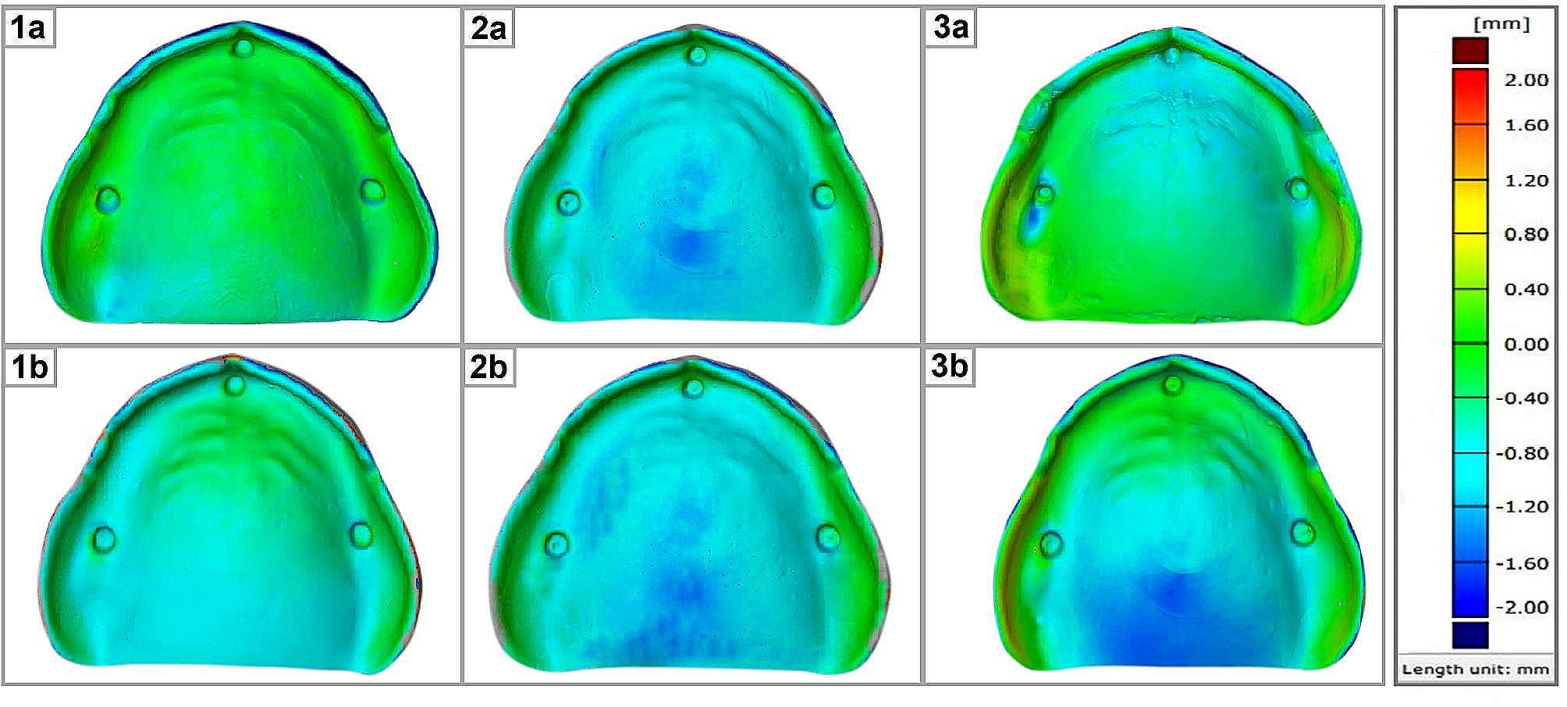
Color maps for different CDBs using superimposition evaluation method, (**1a**) CAD-CAM milled CDBs pre-thermocycling, (**1b**) CAD-CAM milled CDBs post-thermocycling, (**2a**) 3D printed CDBs pre-thermocycling, (**2b**) 3D printed CDBs post-thermocycling, (**3a**) Conventional compression molded CDBs pre-thermocycling, (**3b**) Conventional compression molded CDBs post-thermocycling

The one-way ANOVA revealed a statistically significant difference (*P* < 0.05) between the different groups before thermocycling. The means and standard deviations of the overall misfit of the tested groups before thermocycling are 0.6 ± 0.023, 0.96 ± 0.032, and 0.93 ± 0.024 for groups 1, 2, and 3, respectively. The TPH test showed statistically significant differences between groups 1 and 2, groups 1 and 3, and groups 2 and 3 (*P* < 0.05), as shown in Fig. 1 (1a, 2a, & 3a).

Furthermore, after thermocycling, the one-way ANOVA revealed a statistically significant difference (*P* < 0.05) between the groups. The means and standard deviations of the overall misfit of the tested groups are 0.67 ± 0.089, 1.06 ± 0.06, and 1.56 ± 0.15 for groups 1, 2, and 3, respectively. The TPH test revealed statistically significant differences between groups 1 and 2, groups 1 and 3, and groups 2 and 3 (*P* < 0.05), as shown in Fig. 1 (1b, 2b, & 3b).

### Region-specific misfit

Regarding group 1, the paired t-test revealed statistically significant differences (*P* < 0.05) with respect to precision for the effect of thermocycling in the posterior palatal seal area only, while no statistically significant differences (*P*˃0.05) were identified in the other areas (anterior ridge crest, posterior ridge crest, maxillary tuberosity, palatal vault, and overall), as shown in Table 2; Fig. 1 (1a & 1b).

**Table 2:** Means and standard deviations (mm), and pair-wise comparisons (using paired T test) of different regions and overall misfit in the term of precision of Group 1 (before and after thermocycling)

	Mean	Std. Deviation	95% Confidence Interval of the Difference		Paired T test
			Lower	Upper	P-value
Anterior ridge crest (Pre-thermocycling)	0.5791	0.17738	-0.27779	0.21197	0.768
Anterior ridge crest (Post-thermocycling)	0.6120	0.18746			
Posterior ridge crest (Pre-thermocycling)	0.6292	0.19272	-0.26917	0.23351	0.876
Posterior ridge crest (Post-thermocycling)	0.6470	0.19818			
Maxillary tuberosity (Pre-thermocycling)	0.6178	0.18924	-0.19014	0.18592	0.980
Maxillary tuberosity (Post-thermocycling)	0.6199	0.18988			
Palatal vault (Pre-thermocycling)	0.5777	0.17696	-0.19017	0.13956	0.736
Palatal vault (Post-thermocycling)	0.6030	0.18471			
Posterior palatal seal (Pre-thermocycling)	0.5963	0.18264	-0.49581	-0.03588	0.028*
Posterior palatal seal (Post-thermocycling)	0.8621	0.26407			
Overall (Pre-thermocycling)	0.6000	0.023	-0.18091	0.04331	0.198
Overall (Post-thermocycling)	0.6688	0.08921			

*; Significant level at *p* ≤ 0.05

Concerning group 2, the paired t-test revealed statistically significant differences (*P* < 0.05) with regard to precision for the effect of thermocycling in the posterior palatal seal area and overall, with no statistically significant differences (*P*˃0.05) in the other areas (anterior ridge crest, posterior ridge crest, maxillary tuberosity, and palatal vault), as shown in Table 3; Fig. 1 (2a & 2b).

**Table 3:** Means and standard deviations (mm), and pair-wise comparisons (using paired T test) of different regions and overall misfit in the term of precision of Group 2 (before and after thermocycling)

	Mean	Std. Deviation	95% Confidence Interval of the Difference		Paired T test
			Lower	Upper	P-value
Anterior ridge crest (Pre-thermocycling)	1.0025	0.27697	-0.19352	0.16770	0.875
Anterior ridge crest (Post-thermocycling)	1.0154	0.28054			
Posterior ridge crest (Pre-thermocycling)	0.9421	0.26028	-0.21999	0.19014	0.873
Posterior ridge crest (Post-thermocycling)	0.9570	0.26440			
Maxillary tuberosity (Pre-thermocycling)	0.9771	0.26996	-0.19611	0.16231	0.836
Maxillary tuberosity (Post-thermocycling)	0.9940	0.27463			
Palatal vault (Pre-thermocycling)	0.9191	0.25392	-0.14641	0.13053	0.900
Palatal vault (Post-thermocycling)	0.9270	0.25612			
Posterior palatal seal (Pre-thermocycling)	0.9593	0.26503	-0.77059	-0.17887	0.005*
Posterior palatal seal (post-thermocycling)	1.4340	0.32749			
Overall (Pre-thermocycling)	0.9600	0.032	-0.19520	-0.01576	0.026*
Overall (Post-thermocycling)	1.0655	0.05977			

*; Significant level at *p* ≤ 0.05

Finally, in group 3, the paired t-test revealed statistically significant differences (*P* < 0.05) regarding precision for the effect of thermocycling in all areas (anterior ridge crest, posterior ridge crest, maxillary tuberosity, palatal vault, posterior palatal seal, and overall), as shown in Table 4; Fig. 1 (3a & 3b).

**Table 4:** Means and standard deviations (mm), and pair-wise comparisons (using paired T test) of different regions and overall misfit in the term of precision of Group 3 (before and after thermocycling)

	Mean	Std. Deviation	95% Confidence Interval of the Difference		Paired T test
			Lower	Upper	P-value
Anterior ridge crest (Pre-thermocycling)	0.9428	0.29931	-0.83830	-0.44418	0.000*
Anterior ridge crest (Post-thermocycling)	1.5840	0.25720			
Posterior ridge crest (Pre-thermocycling)	0.9603	0.30487	-1.04650	-0.36895	0.001*
Posterior ridge crest (Post-thermocycling)	1.6680	0.31955			
Maxillary tuberosity (Pre-thermocycling)	0.8966	0.28466	-0.87940	-0.42141	0.000*
Maxillary tuberosity (Post-thermocycling)	1.5470	0.27569			
Palatal vault (Pre-thermocycling)	0.9196	0.29196	-1.00088	-0.23593	0.005*
Palatal vault (Post-thermocycling)	1.5380	0.36207			
Posterior palatal seal (Pre-thermocycling)	0.9308	0.29551	-0.90140	-0.21105	0.005*
Posterior palatal seal (Post-thermocycling)	1.4870	0.35389			
Overall (Pre-thermocycling)	0.9300	0.024	-0.79403	-0.47557	0.000*
Overall (Post-thermocycling)	1.5648	0.15087			

*; Significant level at *p* ≤ 0.05

## Discussion

While many CDB materials are currently available, little data exists regarding their dimensional stability after exposure to oral temperatures, the oral environment, and the temperatures of drinks. In this regard, an in vitro study was conducted herein to evaluate the effect of thermocycling on the trueness and precision of CAD-CAM milled, 3D-printed, and conventional compression molded CDBs.

In the current study, a single metal model was used for the construction of 30 CDBs of different groups. A titanium metal model rather than a stone model was used due to its superior resistance to temperature during the repeated (10 times) compression-molding heat-curing process for the construction of the 10 CDBs of group 3. The null hypotheses stating that there are no significant differences in (i) the trueness between the CAD-CAM milled, 3D-printed, and conventional compression molded CDBs before and after thermocycling and (ii) the effect of thermocycling on the precision within each CDB type were rejected.

To detect the most affected area due to thermocycling and the discrepancies in each tested CDB type and to evaluate the region-specific mismatches, the master cast surface was divided into five functionally relevant sections (anterior ridge crest, posterior ridge crest, maxillary tuberosity, palatal vault, and posterior palatal seal).

Clinicians consider CDB accuracy to be one of the most important features in prosthesis construction approaches [4]. Since the start of PMMA usage in the creation of CDBs, dimensional inaccuracy due to polymerization shrinkage has been inevitable [3]. The accuracy, retention, and stabilization of dentures are impacted by polymerization shrinkage, which also affects patients’ satisfaction and quality of life [1].

The results of the current study reveal a statistically significant difference (*P* < 0.05) between the trueness of the different CDBs before and after thermocycling. In addition, thermocycling had a significant effect on the precision within each type of tested CDB (*P* < 0.05).

The findings reveal that the trueness of the CAD-CAM milled group is significantly higher before and after thermocycling than that of the 3D-printed and traditional compression molded groups (*P* < 0.05). This could be due to using the 5-axis CAD-CAM milling machine, though the 3D printing was carried out with a DLP 3D printer (applying a thickness of 100-µm/layer) and only 3-axis machining. Therefore, the CAD-CAM milled CDBs exhibited greater dimensional accuracy than the 3D-printed ones [15]. Other possible explanations regarding the case of the 3D-printed CDBs include the material constitution, fabrication technique in consecutive layers, and enhanced water sorption with thermal stress [22].

Regarding the precision and region-specific misfit of the CAD-CAM milled and 3D-printed groups, the posterior palatal seal was the only area significantly affected by thermocycling (*P* < 0.05); however, all areas (anterior ridge crest, posterior ridge crest, maxillary tuberosity, palatal vault, posterior palatal seal, and overall) tested for the conventional compression molded CDBs were significantly affected by thermocycling (*P* < 0.05). This may be due to the lower degree of polymerization, weak double bond conversion, and excessive residual monomers that were possibly affected by thermal changes and water sorption, potentially leading to changes in the mechanical properties and dimensional accuracy [23–25]. In addition, water absorption by the acrylic resin could cause the subsequent leaching out of plasticizers and the breakage of resin material [26].

The precision of the posterior palatal seal area was the most significantly affected by thermocycling (*P* < 0.05) for all tested CDB types. On the other hand, the anterior ridge crest, posterior ridge crest, maxillary tuberosity, and palatal vault areas were significantly affected by thermocycling only in the conventional compression molded CDBs (*P* < 0.05). This result agrees with that of Steinmassl et al. [5].

Investigations on the trueness and precision of digitally fabricated CDBs have revealed that, according to the error (i.e., the difference between the surface of the design and the surface of the manufactured product), digitally fabricated CDBs are not inferior to conventional PMMA CDBs. Furthermore, they consistently fall within the clinical tolerance limit [27–30].

In the present study, the variations detected for all the tested CDB materials using different techniques were within the clinically acceptable value (less than 1 mm) [6]. According to prior studies comparing the accuracy of the CAD-CAM milling, injection molding, and compression molding techniques, the first demonstrates noticeably fewer dimensional variations than the remaining two techniques. These findings are consistent with those of the current study [31].

In accordance with the findings of our investigation, Sykora et al. [2] stated that the fabrication methods used to create maxillary CDs have an impact on the fit of a CDB. The CD’s misfit depends on the measurement region. This has been supported by the findings of Goodacre et al. [10], who reported that in the fabrication of maxillary CDs at the edge of the denture border; 6 mm away from the denture border; and in the areas of the crest of the alveolar ridge, palate, and posterior palatal seal, the CAD-CAM milling method shows a lower degree of misfit compared with the molding technique. Furthermore, Steinmassl et al. [5] demonstrated that the alveolar ridge and palate regions in almost all CAD-CAM manufacturing systems show the highest degree of accurate fit, whereas the anterior, lateral, and posterior seal regions show the highest degree of misfit. In a different study, 3D-printed CDBs seemed more accurate in the peripheral seal area, though milled CDBs gave a better fit overall and in the primary stress-bearing areas. However, the accuracy of milled and 3D-printed CDBs in this study was at a clinically acceptable level [32], and in accordance with the findings of the present study, Oğuz et al. concluded that milled CDBs show better adaptation than conventionally fabricated 3D-printed CDBs and that PMMA milling is a repeatable technique allowing for the construction of accurate dentures. They also stated that “clinicians should be cautious about the palatal gap when the compression molding technique is used” [33]. The differences in the results of the current work and other studies may be due to differences in the materials or methodology used.

The study’s conclusions support those of Emam et al., who found that duplicating CDs with the CAD-CAM milling technique yields superior fitting surface accuracy than the 3D printing and injection-molding techniques. These findings indicate that CAD-CAM milling can represent an excellent method for CDB replication, more efficient than the 3D printing and conventional testing techniques. However, in a clinical setting, this distinction might not be apparent [34].

The study’s limitations include a lack of simulations in relation to long-term water storage and oral circumstances (a wet environment), salivary contents, and various beverages. Additionally, using various printing resins and printers may have led to differences in the output. Furthermore, no clinical trials were conducted to test the results in terms of plaque accumulation and sore spots. Therefore, additional research is required to determine how wet conditions and long-term water storage affect the precision and mechanical and physical characteristics of conventional and digitally manufactured denture bases.

## Conclusion

Considering the study’s findings and limitations, we concluded that the trueness of the CAD-CAM milling technique in CD production is higher than that of the 3D printing and conventional compression molding systems both before and after thermocycling. Thermocycling has a significant impact on the precision of the milled CAD-CAM, 3D-printed, and conventional compression molded CDBs. In terms of trueness and precision, thermocycling has the greatest negative impact on the conventional compression molding technique with regard to CD manufacturing. Additionally, among other locations within each group, the posterior palatal seal is the most negatively affected area by thermocycling.

## Data Availability

The data will be available on reasonable request from the corresponding author.
